# Art for a Lifetime

**DOI:** 10.1093/geroni/igaa057.2964

**Published:** 2020-12-16

**Authors:** Alexis Schramel

**Affiliations:** Upper Iowa University, Monona, Iowa, United States

## Abstract

Art for a lifetime was a bi-weekly programming opportunity in a long-term care (LTC) community taught by students and faculty. We predicted that 1) Resident physical and mental abilities may influence art-making preferences and 2) residents would be concerned about their perceived lack of creativity. Findings revealed that residents with arthritis preferred working with larger forms (e.g., collage and sculpture) and residents with dementia preferred working with bright, colorful materials. Private one-on-one sessions were beneficial for increasing resident confidence and for working on individual projects. Programming also allowed for increased social opportunities among residents, offering occasions to reflect on life experiences. Overall, facilitators of art programming need to quickly adjust and adapt programming based on resident abilities and preferences. Expanding art programming to other long-term care facilities is important for providing increased opportunities for autonomy and decision making, areas that often become more limited when living in LTC.

